# Innovative nanocomposite of intercalated alginate on nanomagnetite@ Al_2_O_3_@nanobentonite for uptake recovery of ^152–154^Eu and ^60^Co radionuclides from nuclear wastewater

**DOI:** 10.1038/s41598-025-09238-x

**Published:** 2025-07-12

**Authors:** Yasser K. Abdel-Monem, M. I. Ayad, Ayman H. Elewa, Elhassan A. Allam, Mohamed A. Gizawy, Rehab M. El-Sharkawy, Mohamed E. Mahmoud

**Affiliations:** 1 Chemistry Department, Faculty of Science, Menoufia University, Shebin El-Koom, Menoufia Egypt; 2 Central Laboratories, Ministry of Health and population, P.O. Box 21518, Alexandria, Egypt; 3 Labeled Compounds Department, Hot Labs Center, Egyptian Atomic Energy Authority, P.O. Box 13759, Cairo, Egypt; 4 Chemistry Department, Faculty of Dentistry, Pharos University in Alexandria, P.O. Box 37, Sidi Gaber, Alexandria Egypt; 5 Faculty of Science, Chemistry Department, Alexandria University, P.O. Box 426, Ibrahimia, Alexandria 21321 Egypt

**Keywords:** ^60^Co and ^152–154^Eu, Nuclear wastewater, Nanobentonite, N-Fe_3_O_4_, N-Al_2_O_3_, Sodium alginate, Adsorptive uptake studies, Environmental sciences, Chemistry

## Abstract

In this study, an innovative nanocomposite has been assembled through the intercalation of alginate onto the surface of nanomagnetite@nano-Al_2_O_3_@nanobentonite, resulting in the formation of Alg@N-Fe_3_O_4_@N-Al_2_O_3_@N-Bent. The developed nanocomposite underwent characterization through various techniques, including FT-IR, TGA, SEM, and XRD to refer to an average particle size at 80.0–90.0 nm with numerous related functional groups of its constituting unites. The evaluation of Alg@N-Fe_3_O_4_@N-Al_2_O_3_ aimed to identify its capacity to uptake and recover two radioactive nuclides, viz. ^60^Co and ^152–154^Eu, from nuclear wastewater. The outcomes of the study revealed that the most favorable conditions for the uptake of ^60^Co and ^152–154^Eu were a pH level of 6.0 and a contact time of 4.0 min. Under these conditions, the maximum uptake capacity values were determined as 82.71 mg g^−1^ (^60^Co) and 180.4 mg g^− 1^ (^152–154^Eu). The uptake process was characterized by fitting the data to *pseudo*-first and *pseudo*-second order models, in addition to this; the ^152–154^Eu nuclide was specifically also fitted to the intra-particle model. The related adsorption isotherm models to the uptake of ^60^Co and ^152–154^Eu were investigated and the findings indicated that ^152–154^Eu nuclide was well-fitted to the Langmuir and Dubinin-Radushkevich (D-R) models, whereas ^60^Co nuclide showed strong alignment with the Langmuir, Temkin, and D-R models. The outlined results validated the effectiveness of the Alg@N-Fe_3_O_4_@N-Al_2_O_3_ nanocomposite in the remediation of polluted nuclear wastewater with the radioactive nuclides ^152–154^Eu and ^60^Co, providing a strong emphasis in minimizing of their risks before being released into the environment.

## Introduction

Nuclear technology is the backbone of diverse industrial applications due to its wide use in nuclear facilities like nuclear power reactors to produce electricity and research nuclear reactors, as well as other applications in nuclear medicine, pharmaceuticals, and agriculture^1^. The most challenging issue in this technology is related to the waste management of radioactive nuclides that are generated from such applications, in addition to waste from fission reactions, mining processes, and other nuclear processes^2^. Water, soil, and air contaminated with diverse radioactive isotopes like ^137^Cs, ^152^Eu, ^60^Co, ^106^Ru, ^125^I, ^129^I, ^131^I, ^99^Tc, ^99^Mo, ^90^Sr, ^241^Am, ^144^Ce, and ^75^Se are targets for recent research to find suitable technological methods to remediate the contaminated environment containing radioactive isotopes^3^. The significant hazardous consequences of these radioactive isotopes are primarily connected to their classification as continuous sources of radiation, which includes alpha, beta, and gamma radiation, along with their potential to accumulate within human tissues^4^. Some of these radiations are known to damage living tissue, alter DNA, and may lead to death according to the time and intensity to which a person is exposed. Therefore, dangerous radioactive isotopes must be removed from nuclear wastewater by using effective methods for each type before being discharged into the environment^5^. The remediation process of contaminated water with radioactive nuclides before discharge may be accomplished by micromembrane and nanomembrane based techniques, ion exchange, and adsorption via applications of diverse materials such as natural and modified clays, polymers, nanometal oxides, and nanocomposites^6–15^. The technique of adsorption is extensively utilized for extracting radionuclides from contaminated water, owing to its economical operation and the fact that it does not produce secondary pollutants during the treatment^6–8^. Numerous nanomaterials have been used in many fields, including health care, electron devices, energy, water decontamination, etc., and the past decades have witnessed increasing applications of nanomaterials in the fields of environmental protection and water remediation^9^. The reason for such interest in nanomaterials is mainly based on their extraordinary adsorption capacity and reactivity properties, both of which favor the removal of radioactive isotopes from wastewater^10^. Therefore, several types of materials have been used to remove radioactive nuclides from wastewater, such as nanometal oxides, natural clays, carbohydrates polymers, Metal Organic Frameworks MOFs and other kinds of nanocomposite materials^11–22^. The removal of cesium using layered MOFs through an ion exchange mechanism has been established^11^. Two-dimensional materials called “Mexenes” have been established for the removal of radioactive nuclides^12^. Bentonite is a type of natural clay known for its significant ability to absorb water, consisting of two tetrahedral layers of silica sandwiched between a single octahedral layer of alumina^13^. The application of bentonite and its diverse nanocomposites has proven effective in serving as adsorbents for the removal of various radioactive nuclides, including ^60^Co, ^152–154^ Eu, and ^65^Zn, from radioactive wastewater^14–16^. Sodium alginate, recognized as a biodegradable polymer, has been examined and utilized in various nanocomposites for the purpose of adsorbing diverse heavy metals as well as radioactive isotopes from wastewater^16,17^. Alginate is a natural polysaccharide polymer that is mostly prepared from brown seaweeds such as ecklonia, laminaria, macrocystis, sargassum, and turbinaria. The study focused on the examination of cross-linked granules composed of alginate and poly(vinyl alcohol) that contained immobilized ferric hexacyanoferrate, specifically for the purpose of adsorbing and removing radioactive ^137^Cs from water^16,17^. Removal of ^152–154^Eu and ^60^Co attracts attention for a lot of researchers in their research work using adsorption techniques and different kinds of nanocomposite materials^18–28^.

An investigation was performed to examine the removal behavior of cesium and europium from contaminated water solutions by means of activated Al_2_O_3_. The activated Al_2_O_3_ was produced via a solution combustion method at temperatures of 200 °C and 400 °C, and the time required for synthesis varies, falling within the range of 10 to 30 min^18^. Sorbents such as MnO_2_, Mn_3_O_4_, and MNOOH were developed and employed to eliminate radionuclides, specifically cobalt, europium, cerium, and strontium, from tap water^19^. Nanocalcium silicate, synthesized via a wet method, was examined for its efficacy as an adsorbent in the extraction of long-lived radionuclides ^152‏−154^Eu and ^134^Cs from aqueous media. The adsorption rate was found to improve with an increase in temperature^20^. Hydroxyl magnesium silicate, a naturally occurring mineral composite, has been reported as an effective natural sorbent capable of adsorbing long-lived radioactive nuclides such as ^152–154^Eu, ^60^Co, and ^134^Cs from aqueous solutions^21^. Moreover, extensive research has been conducted on the removal processes for ^152–154^Eu and ^60^Co^22–28^.

In this context, nanobentonite (N-Bent) which is a natural clay with high thermal and mechanical stability, was intercalated with magnetic iron oxide (N-Fe_3_O_4_) and nanoalumina (N-Al_2_O_3_) and further functionalized with sodium alginate using 1,2-dichloroethane as a linker to form a nanocomposite Alg@N-Fe_3_O_4_@N-Al_2_O_3_. The metal oxides N-Fe_3_O_4_ and N-Al_2_O_3_ were prepared via the co-precipitation approach and the combustion approach, respectively. Various techniques were employed to characterize the novel nanocomposite, which was then applied to eliminate the targeted radioactive isotopes ^152–154^Eu and ^60^Co from nuclear wastewater. The optimum conditions for adsorptive uptake of ^152–154^Eu and ^60^Co radioactive nuclides from radioactive wastewater were studied through diverse experimental control parameters.

## Experimental

### Materials and instrumentations

This research involved the procurement and use of all chemicals in their unrefined state, without undergoing any purification processes. The specifications for these chemicals can be found in Table 1, and the details regarding the instruments employed are provided in Table 2.

**Table 1:** Chemicals’ purity and materials’ specifications.

Chemical name	MF	FW (g/mol)	Assay (%)	Company
Aluminum carbonate	Al_2_(CO_3_)_2_	243.88	99.0	Alfa Aesar, UK
Glycine	NH_2_-CH_2_-COOH	75.07	99.0	Oxford, India
Urea	CO(NH_2_)_2_	60.06	99.0	Oxford, India
Ferric chloride hexahydrate	FeCl_3_·6H_2_O	270.30	98.0	Alpha chemika, India.
Iron (II) sulphate hepta hydrate	FeSO_4_·7H_2_O	278.01	98.0	Nice, India.
Sodium hydroxide	NaOH	40	99.0	Sigma Aldrich, USA
Nano bentonite	H_2_Al_2_SiO_6_	180.1	95.0	Sigma Aldrich, USA
Sodium alginate	C_6_H_9_NaO_7_	216.12	91.4	Alfa Aesar, UK

**Table 2:** Instrumentation used for characterization of the prepared Alg@N-Fe_3_O_4_@N-Al_2_O_3_@N-Bent nanocomposite.

Instrument	Model	Data	Conditions	Technique
Fourier-transform infrared spectro-photometer (FT-IR)	BRUKER Tensor 37	FT-IR spectrum	400–4500 cm^−1^	Using KBr pellets
TGA-7 thermobalance	A Perkin-Elmer	Thermogram	Temperature heating (25–800 °C)	Pure nitrogen atmosphere, flow rate = 40 mL/min, heating rate = 10 °C/min and sample mass in the range of 5.0–6.0 mg
X-ray diffraction (XRD)	Shimadzu lab x 6100, Kyoto, Japan	XRD spectrum	40 kV, 30 mA, λ = 1 Å, 2θ from 10 to 80, recording steps of the diffraction data of 0.02°, at a time of 0.6 s, at room temperature (25 °C).	X-ray diffractometer, using target Cu-Kα
Scanning electron microscope (SEM)	JSM-lT200, JEOL Ltd	SEM images	Imaging mode	Sputtering coating (JEOL-JFC-1100E)

### Synthesis of the Alg@N-Fe_3_O_4_@N-Al_2_O_3_@N-Bent nanocomposite

In this work, two nano-metal oxides were produced through combustion and co-precipitation synthesis techniques, resulting in N-Al_2_O_3_ and N-Fe_3_O_4_. These nano-metal oxides were subsequently intercalated with nanobentonite and modified using sodium alginate.

#### Synthesis of nanomagnetite (N-Fe_3_O_4_)

A co-precipitation approach was implemented to synthesize nano magnetite by facilitating the reaction between ferric ions and ferrous ions, with sodium hydroxide solution being added gradually. The reaction proceeded as follows: 4.0 g of ferrous sulfate heptahydrate (FeSO_4_·7H_2_O) and 6.0 g of ferric chloride hexahydrate (FeCl_3_·6H_2_O) were mixed and dissolved in 200 mL of water. The subsequent step involved the gradual addition of 40.0 mL of 6.5 M sodium hydroxide into the aforementioned mixture, accompanied by vigorous stirring at a temperature of 60 °C for 70 min. Ultimately, a black precipitate (N-Fe_3_O_4_) was formed, which was then washed multiple times with deionized water until pH 7.0 was reached. The produced precipitate was subsequently dried at 60 °C for a period of 6 h^29^.

#### Synthesis of Nanoalumina (N-Al_2_O_3_)

Nanoalumina particles were produced through a combustion synthesis technique, employing 1.0 g of aluminum carbonate (Al_2_(CO_3_)_3_) and 1.0 g of glycine in a weight ratio of 1:1. The components were thoroughly homogenized and afterward placed in a muffle furnace at a temperature of 700 °C for a duration of 5.0 h, resulting in the formation of a white precipitate identified as N-Al_2_O_3_^30^.

#### Intercalation of nanobentonite with N-Fe_3_O_4_ and N-Al_2_O_3_

A total of 2.0 g of nanobentonite was introduced to 150.0 mL of 0.001 M NaOH to facilitate the activation of the bentonite clay and enhance the intercalation process. Subsequently, 1.0 g of N-Fe_3_O_4_ and 1.0 g of N-Al_2_O_3_ were added to the bentonite gel solution, which was then subjected to stirring and heated to 80 °C. The resulting nanocomposite, known as magnetic-alumina bentonite (N-Fe_3_O_4_@N-Al_2_O_3_@N-Bent), underwent multiple washings with deionized water to ensure the complete removal of residual sodium hydroxide and subsequently dried at 60 °C for a period of 6 h^14,15^.

#### Functionalization of N-Fe_3_O_4_@N-Al_2_O_3_@N-Bent with sodium alginate

**Scheme 1:**
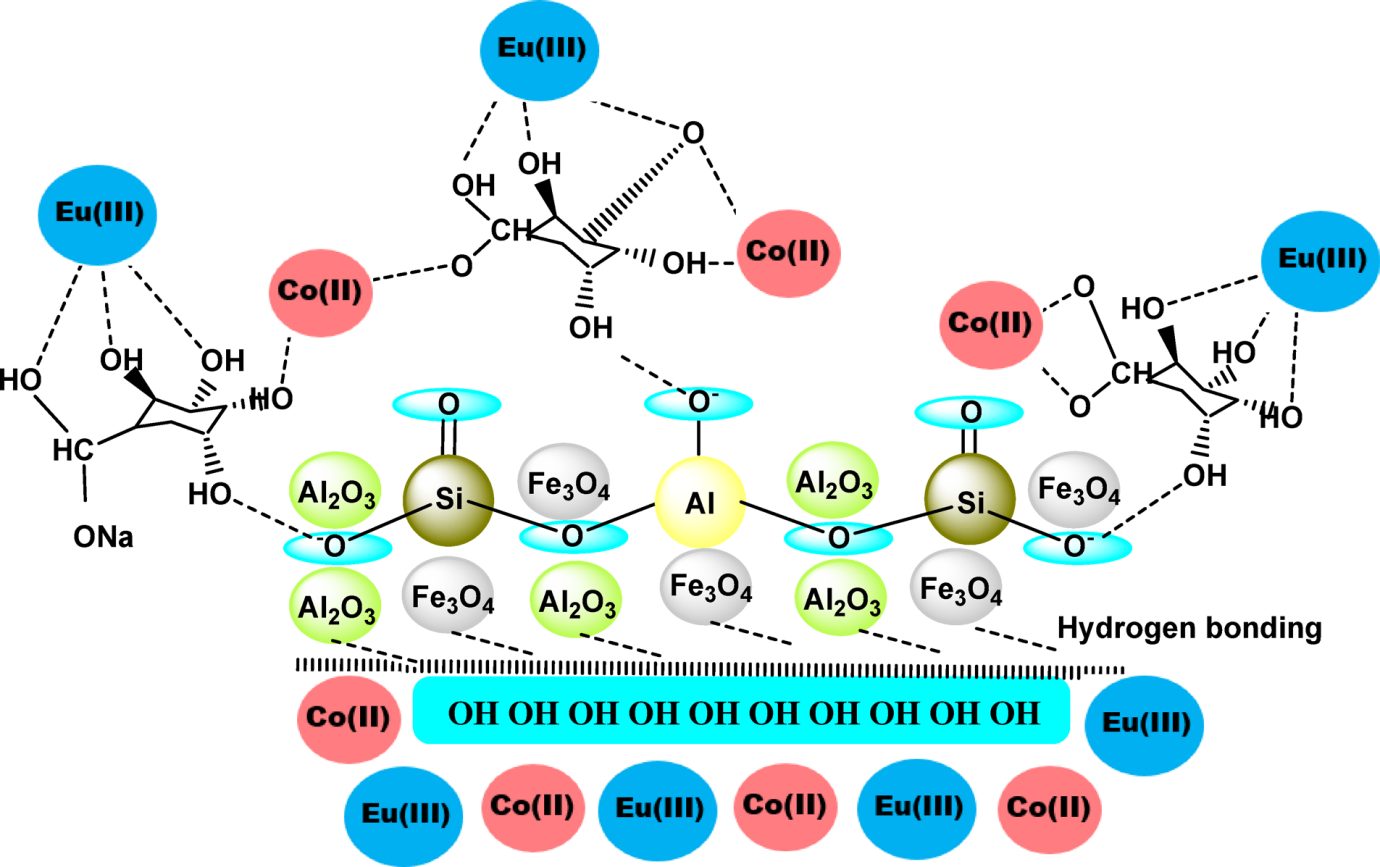
The interaction between the different functional groups of the Alg@N-Fe_3_O_4_@N-Al_2_O_3_@N-Bent nanocomposite and the radioactive nuclides ^152-154^Eu and ^60^Co.

In the preparation process, 2.0 g of N-Fe_3_O_4_@N-Al_2_O_3_@N-Bent were combined with 1.0 g of sodium alginate. An excess of 1,2-dichloroethane (20 mL) was then added to a round-bottom flask, which was subjected to reflux at 80 °C for 6 h while being stirred vigorously. The outcome of this process was the Alg@N-Fe_3_O_4_@N-Al_2_O_3_ nanocomposite, which appeared as a grey powder featuring smooth particles^16^. The interaction between the different functional groups of Alg@N-Fe_3_O_4_@N-Al_2_O_3_ with ^152–154^Eu (III) and ^60^Co (II) radionuclides is supposed to occur according to Scheme 1.

### Preparation of radioactive ^60^Co and ^152–154^Eu nuclides

^60^Co and ^152–154^Eu radioactive nuclides were prepared by the irradiation of 20.0 mg of the salts CoCl_2_·6H_2_O, and Eu_2_O_3_, respectively. Each of the two salts was individually encased in aluminum foil before being placed into an aluminum can intended for irradiation for a duration of approximately 48 h at the Egyptian Second Research Reactor (ETRR-2) located in Inshas, Egypt. Following an appropriate cooling period, both irradiated salts were dissolved in 10.0 mL of HCl (1.0 mol L^−1^), which was designated as the stock solution. This solution was then stored and employed in all subsequent adsorption experiments^16^.

### Remediation of ^60^Co and ^152–154^Eu radioactive nuclides onto Alg@N-Fe_3_O_4_@N-Al_2_O_3_@N-Bent

The adsorption behavior of radioactive nuclides ^60^Co and ^152–154^Eu onto the Alg@N-Fe_3_O_4_@N-Al_2_O_3_ nanocomposite was investigated through batch technique, taking into account various experimental controlling variables including the contact time, the initial concentration of the radioactive nuclides, pH levels, and temperature of the reaction.

### Batch adsorption study

An amount of 10.0 mg of the Alg@N-Fe_3_O_4_@N-Al_2_O_3_ nanocomposite was introduced to a 10.0 mL glass vial. Afterward, 5.0 mL of a simulated radioactive solution was spiked with ^152–154^Eu and ^60^Co radioactive nuclides, with pH values varied from 1.0 to 6.0 as per the experimental design. The duration of shaking was maintained at 4.0 h with a thermostatically controlled shaker operating at 140 rpm and a temperature of 25 °C. Upon completion of the equilibration process, a 1.0 mL portion was taken from the resulting aqueous solution. The total radioactivity of the radioactive isotopes ^152–154^Eu(III) and ^60^Co(II) was then individually quantified using sodium iodide in a single-channel analyzer (Spetech ST 360 to Crystal, USA), taking advantage of their unique γ-rays energies.

The determination of both the metal capacity (q_e_) in mg g^−1^ and the removal percentage (R%) for the radioactive isotopes ^60^Co(II) and ^152–154^Eu(III) was conducted through the application of Eqs. (1) and (2)^26–28^.

1 $$\:{\text{R}}_{{\%}}=\frac{{\text{C}}_{\text{o}}-{\text{C}}_{\text{e}}}{{\text{C}}_{0}}\times 100$$

2 $$\:{\text{q}}_{\text{e}}=\frac{{\text{C}}_{\text{o}}-{\text{C}}_{\text{e}}}{\text{m}}\times\:\text{v}\:\:$$

Where, C_o_ is defined as the initial concentration, while C_e_ is the final concentration of the radionuclides ^60^Co(II) and ^152–154^Eu (III) expressed in mg/L. Here, V is used to indicate the volume of the solution in liters, whereas m represents the mass of the Alg@N-Fe_3_O_4_@N-Al_2_O_3_ nanocomposite, measured in grams.

By the same way the different parameters have been studied at various reaction times (0.5, 1.0, 1.5, 2.0, 2.50, 3.0, 3.50, 4.0 and 24.0 min) and the reaction temperature was studied at diverse values (20, 40, 60, 80 °C). On the other hand, the point of zero charge (PZC) for Alg@N-Fe_3_O_4_@N-Al_2_O_3_ was evaluated at various pH values (1.0–7.0) through the pH-drift method as documented previously^16^. In a standard procedure, multiple 100 mL conical flasks were filled with 0.01 M NaCl (50 mL) solution each. The pH of these solutions was adjusted from 1.0 to 7.0, using either 0.1 M NaOH or 0.1 M HCl. In every NaCl solution with a predetermined initial pH (pH_initial_), 10.0 mg of Alg@N-Fe_3_O_4_@N-Al_2_O_3_ was introduced. Each mixture was shaken at a speed 250 rpm for 4 h at 25 °C and allowed to sit overnight. The final pH measurements (pH_final_) were subsequently documented and the variation between the initial and final pH values (ΔpH = pH_final_ - pH_initial_) was computed and represented graphically against the initial pH to determine the PZC at ΔpH = 0.

## Results and discussion

### Characterization of the Alg@N-Fe_3_O_4_@N-Al_2_O_3_@N-Bent nanocomposite

#### XRD analysis

**Fig. 1:**
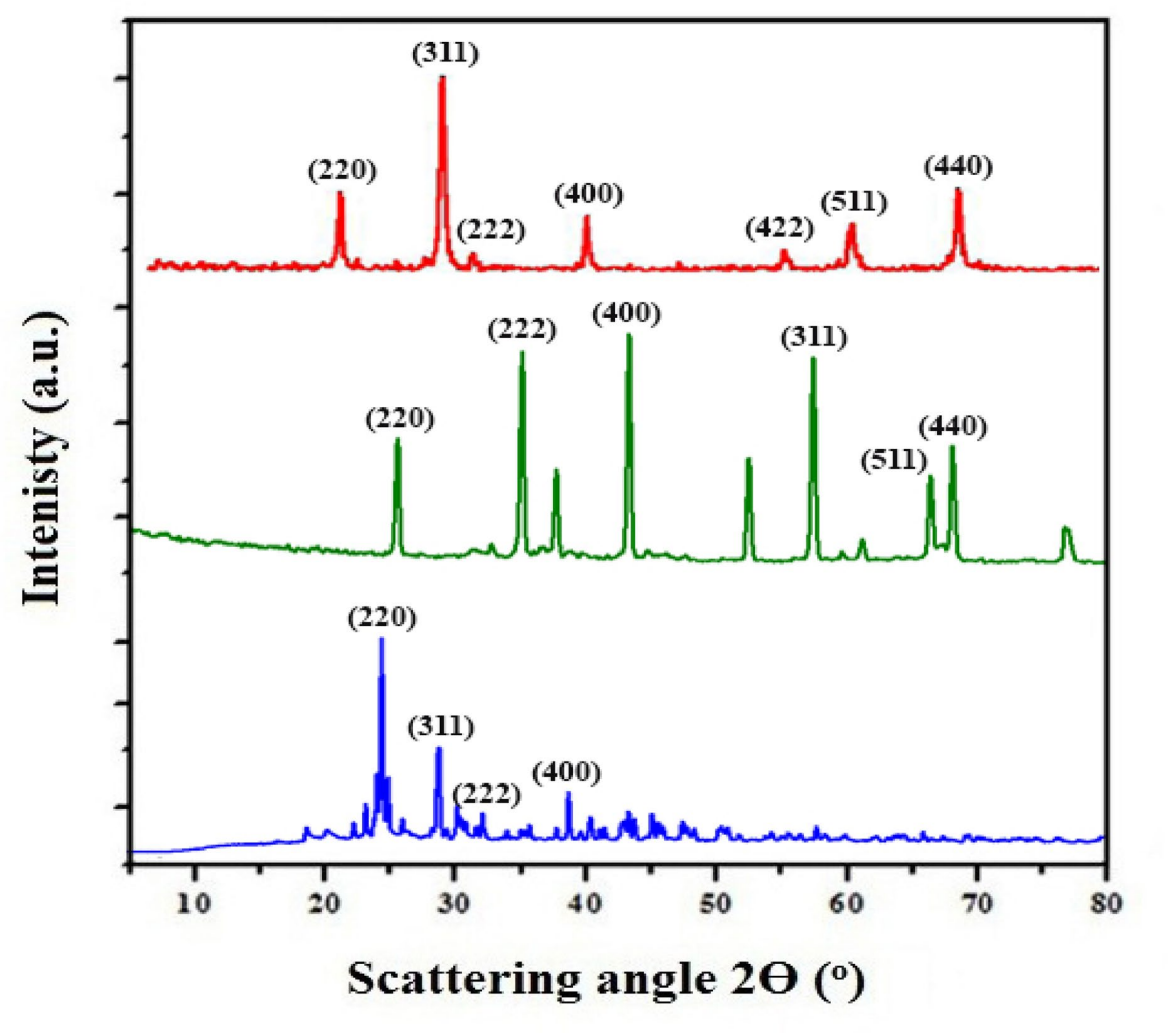
XRD patterns of (**a**) N-Fe_3_O_4_, (**b**) N-Al_2_O_3_ and **(c)** The Alg@N-Fe_3_O_4_@N-Al_2_O_3_@N-Bent nanocomposite.

Figure 1 presents the X-ray diffraction patterns of N-Fe_3_O_4_, N-Al_2_O_3_, along with the corresponding Alg@N-Fe_3_O_4_@N-Al_2_O_3_ nanocomposite. In Fig. 1a, the XRD diffraction pattern of N-Fe_3_O_4_ is depicted, featuring seven notable diffraction peaks at 2θ = 30.33°, 34.48°, 36.13°, 42.11°, 53.82°, 58.71°, and 63.26°. These peaks are associated with the (220), (311), (222), (400), (422), (511), and (440) planes of the spinel crystal structure, which is typical for magnetite nanoparticles that demonstrate well-defined crystallinity (magnetite, JCPDS card no. 85-1436)^31,32^. No significant XRD peaks that suggest impurities are present, indicating that N-Fe_3_O_4_ is produced with a high degree of purity. Distinct peaks in the XRD pattern of N-Al_2_O_3_ is clearly recognized, as shown in Fig. 1b. These peaks are found at 2θ = 35.28°, 37.43°, 43.27°, 57.11°, 61.92°, and 66.43°. The relevant Miller indices for the Bragg’s peaks are (220), (222), (400), (311), (511), and (440) planes, respectively and these are representative of aluminum oxide having a cubic face-centered structure^30^.

On the other hand, Fig. 1c displays the X-ray diffraction pattern of Alg@N-Fe_3_O_4_@N-Al_2_O_3_. Upon examining the diffractogram, it is evident that several XRD peaks are present, which correspond to N-Fe_3_O_4_, N-Al_2_O_3_, alginate, and N-Bent, appearing at their anticipated locations and consistent with their respective amounts. The prominent peaks appear at 2θ values of 21.9°, 26.2°, 27.8°, 36.3°, 37.6°, and 54.4°, corresponding to the aluminum silicate framework identified in bentonite^14,15^. The low-intensity peaks identified at 2θ values of 12.78°, 14.32°, 18.10°, 22.54°, 24.77°, and 28.71° are associated with the alginate segment of the nanocomposite sample, reflecting its amorphous nature^16^. Additionally, the peak identified at 2θ = 46.0° is associated with N-Al_2_O_3_^30^. The prominent peaks observed at 26.15° and 33.20° are attributed to the presence of aluminium silicate and N-Fe_3_O_4_, respectively^33^. These findings confirm the successful intercelation of both N-Fe_3_O_4_ and N-Al_2_O_3_ nanoparticles into the N-Bent clay matrix, along with the surface modification with sodium alginate.

#### FT-IR analysis

**Fig. 2:**
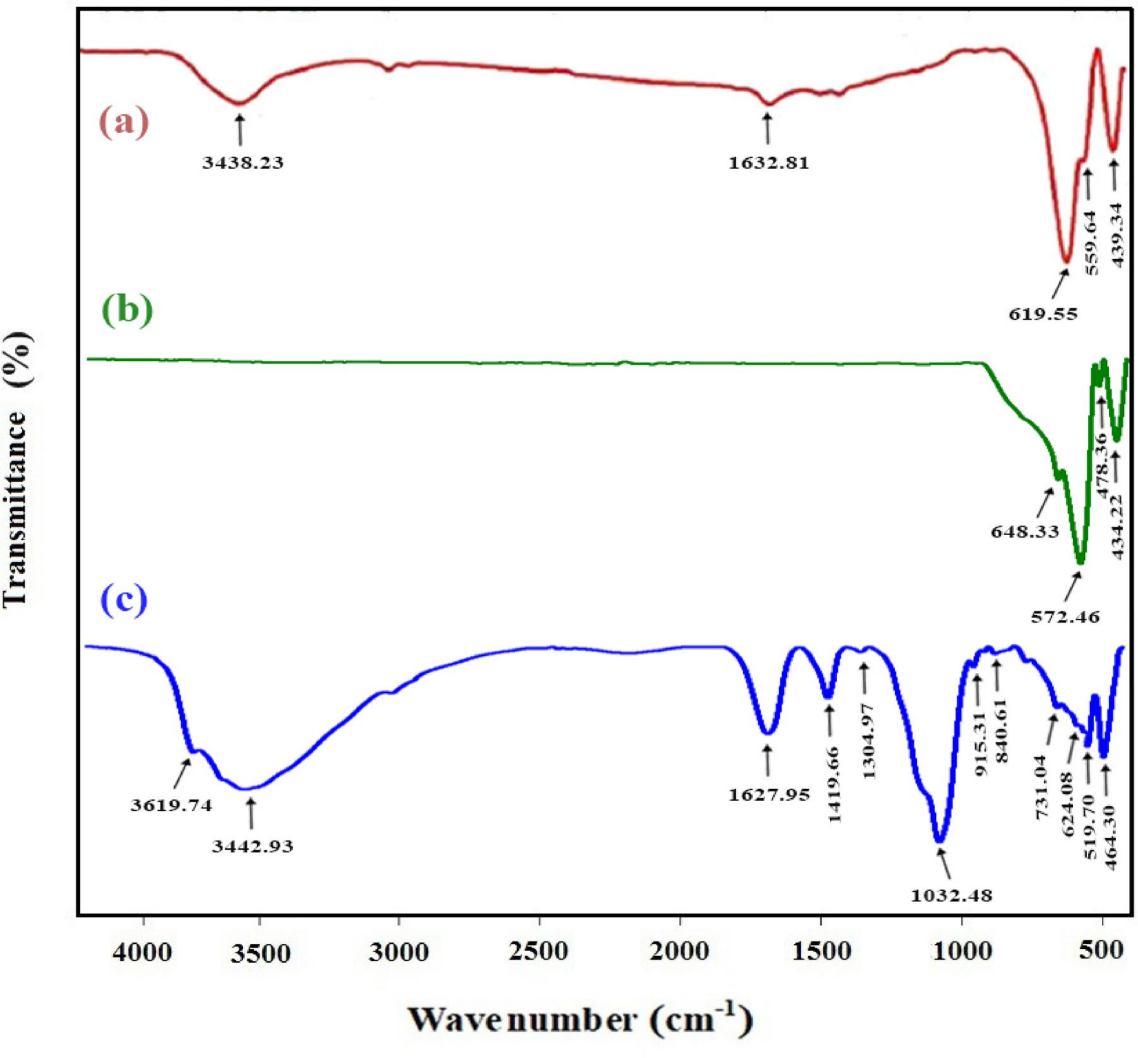
FT-IR spectrum of (**a**) N-Fe_3_O_4_, (**b**) N-Al_2_O_3_ and (**c**) The Alg@N-Fe_3_O_4_@N-Al_2_O_3_@N-Bent nanocomposite.

The synthesized N-Fe_3_O_4_, N-Al_2_O_3_, as well as the Alg@N-Fe_3_O_4_@N-Al_2_O_3_ nanocomposite underwent examination and was represented through FT-IR spectral analysis across the wavelength range from 400 to 4500 cm^−1^. The FT-IR spectrum of N-Fe_3_O_4_ is depicted in Fig. 2a, which identifies five key peaks at 439.34, 559.64, 619.55, 1,632.81, and 3,438.23 cm^−1^. The peaks at 439.34, 559.64, and 619.55 cm^−1^ are attributed to the stretching vibration mode of Fe–O bonds^32^. The band at 1,632.81 cm^−1^ is associated with the bending vibrational modes of H–O–H in water molecules^33^. The peak at 3,438.23 cm^−1^ corresponds to the streaching vibration of the O–H group^33^. With respect to N-Al_2_O_3_ (Fig. 2b), four distinct peaks were recognized at 434.22, 478.36, 572.46, and 648.33 cm^−1^, which are associated with the Al–O–Al stretching vibration^18^.

The FT-IR spectrum of Alg@N-Fe_3_O_4_@N-Al_2_O_3_ is illustrated in Fig. 2c, where it is evident that it displays multiple prominent peaks corresponding to all the functional groups of the nanocomposite components, accompanied by a minor shift in wavenumber. The peaks observed at 519.70 and 840.61 cm^−1^ can be attributed to the intercalation of nanomagnetite with nanobentonite, which is a consequence of the asymmetric and symmetric vibrations of the Fe–O bond^31–33^. The peak located at 731.04 cm^−1^ associated with the vibration of Al–O in nano Al_2_O_3_ along with the out-of-plane vibrations of both Al–O and Si–O found in nanobentonite^30^. The carbohydrate structure of sodium alginate is represented by the three peaks identified at 464.30, 624.08, and 915.31 cm^−1^. Additionally, the weak bands noted at 1,419.66 and 1,304.97 cm^−1^ are related to the COO^−^ group, while the medium peak detected at 1,627.95 cm^−1^ corresponds to the C = O functional group within the sodium alginate backbone. The broad peak identified at 3442.93 cm^−1^ is indicative of hydroxyl groups present in the molecular chains of sodium alginate^16^. The prominent peak observed at 1,032.48 cm^−1^ is indicative of the (OH)–Al–O asymmetric stretching vibration associated with nanobentonite, providing substantial evidence for the existence of a silicate structure within bentonite clay^14–16^. Moreover, the peak detected at 519.70 cm^−1^ is linked to the existence of octahedral Si–O–Al as well as the bending vibrations of Si–O–Si. The identified peak at 3619.74 cm^−1^, conversely, relates to the existence of water molecules located on the surface of the nanocomposite.

#### TGA analysis

**Fig. 3:**
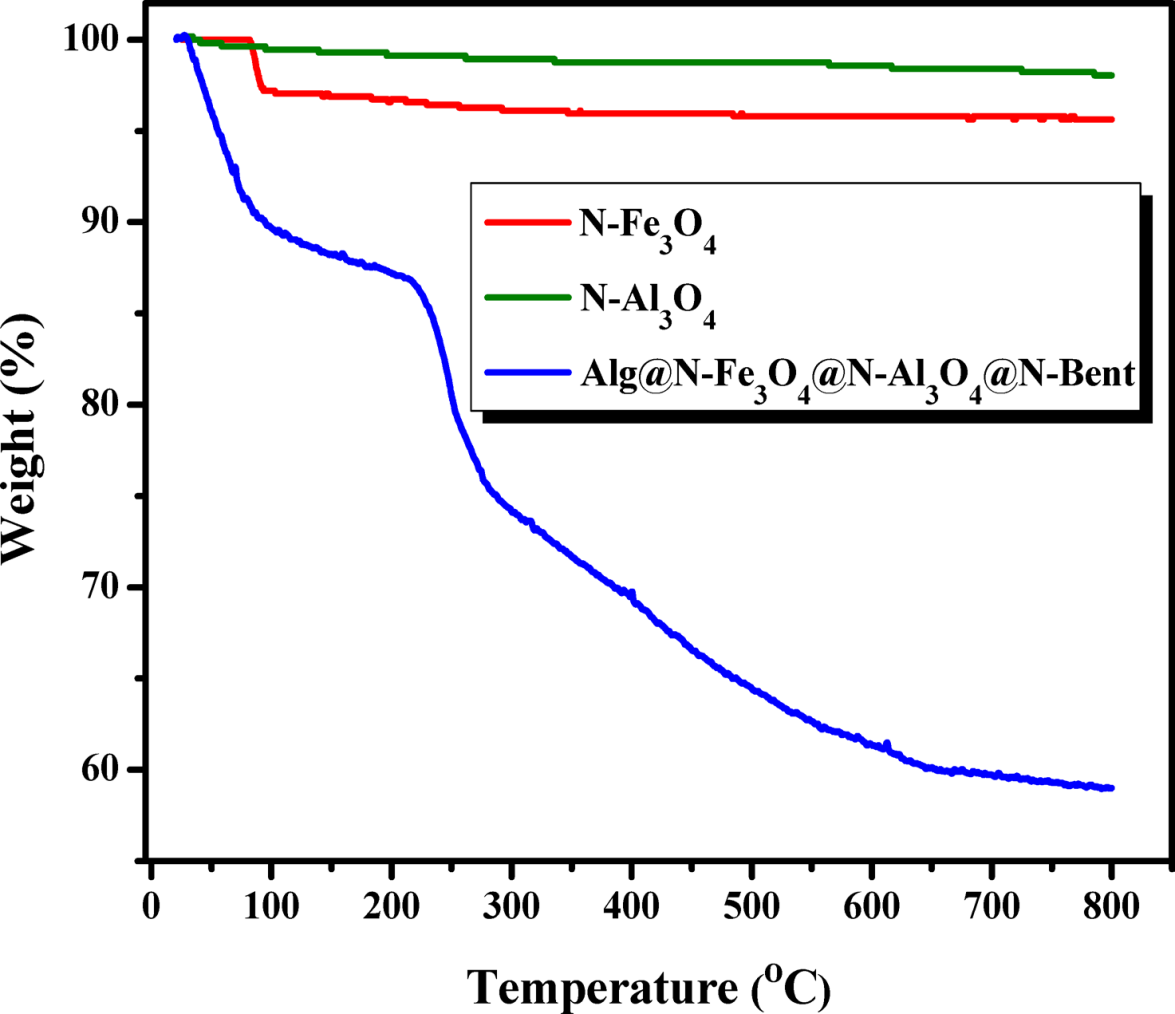
TGA thermogram of N-Fe_3_O_4_, N-Al_2_O_3_ and the Alg@N-Fe_3_O_4_@N-Al_2_O_3_@N-Bent nanocomposite.

Figure 3 presents the TGA thermograms of N-Fe_3_O_4_, N-Al_2_O_3_, along side with the synthesized Alg@N-Fe_3_O_4_@N-Al_2_O_3_@N-Bent nanocomposite, providing a comprehensive understanding of their thermal stability. From the analysis of the thermograms of N-Fe_3_O_4_ and N-Al_2_O_3_, it is evident that both materials demonstrated significant thermal stability. They only lost 1.79% and 1.35% of their original mass, respectively, when subjected to temperatures up to 800 °C, and exhibited a single mass loss peak beginning at 120 °C. This degradation peak corresponds to the evaporation of water that was physically adsorbed onto the surfaces of the synthesized nanopowders^18,33^. Conversely, the thermogram obtained from TGA analysis of Alg@N-Fe_3_O_4_@N-Al_2_O_3_@N-Bent is marked by various stages of thermal degradation. The first stage, occurring within the temperature range of 22.69 to 143.20 °C, shows an 11.77% weight loss, primarily due to the loss of water molecules that were adsorbed on the surface of the nanocomposite, along with some organic fragments of sodium alginate. The second stage, which takes place from 143.20 to 339.93 °C, results in a 16.23% weight loss, linked to the further thermal breakdown of sodium alginate as the alginate rings begin to crack^16^. The third stage is identified between 339.93 and 597.11 °C, with a weight loss of 10.79%, which pertains to the degradation of the benzene ring. The final step is recognized at a temperature range of 597.11–799.61 °C, where a degradation loss of 2.37% is noted, resulting from the breakdown of certain ash constituents in the bentonite intercalated with oxides. The overall degradation across all steps totals 41.16%, indicating the successful application of the synthesis approach for the nanocomposite, as referenced in the experimental “Materials and instrumentations ” section.

#### SEM of Alg@N-Fe_3_O_4_@N-Al_2_O_3_@N-Bent

**Fig. 4:**
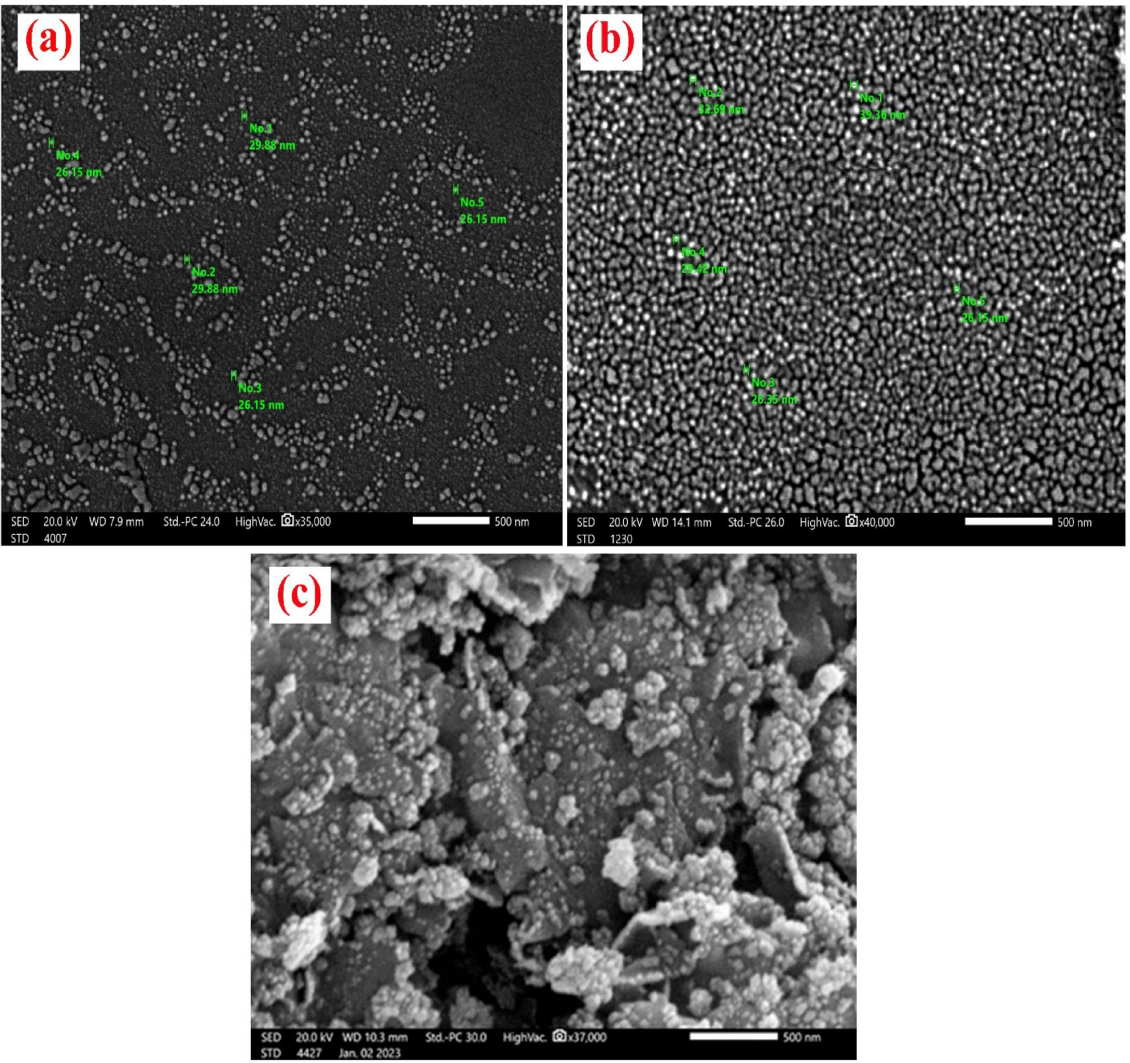
SEM of (**a**) N-Fe_3_O_4_, (**b**) N-Al_2_O_3_ and **(c)** The Alg@N-Fe_3_O_4_@N-Al_2_O_3_@N-Bent nanocomposite.

The SEM images representing N-Fe_3_O_4_, N-Al_2_O_3_, and the Alg@N-Fe_3_O_4_@N-Al_2_O_3_@N-Bent nanocomposite are illustrated in Fig. 4. As illustrated in Fig. 4a, the SEM image of the synthesized N-Fe_3_O_4_ indicates that the nanoparticles are nearly spherical, with a narrow distribution in particle sizes. The average particle size was estimated around 27.64 nm. The SEM image of N-Al_2_O_3_ (Fig. 4b) reveals clusters of individual alumina particles. A detailed examination of the agglomerated mass shows distinct aggregates of the nanoparticles. Within these clusters, individual nanoparticle crystals can be identified. They appear powdery, dense, uniform, spherical in shape with an average particle size about 30.59 nm. In contrast, the SEM image of Alg@N-Fe_3_O_4_@N-Al_2_O_3_@N-Bent (Fig. 4c) reveals the presence of nanobentonite sheets that are interlinked with sodium alginate polysaccharides network, while the intercalated alumina and magnetite nanoparticles appear as beads dispersed within the sheets.

### Adsorptive removal of ^152−54^Eu and ^60^Co radioactive nuclides by Alg@N-Fe_3_O_4_@N-Al_2_O_3_@N-Bent

#### The impact of pH level on the adsorption process and point of zero charge (pH_zpc_)

**Fig. 5:**
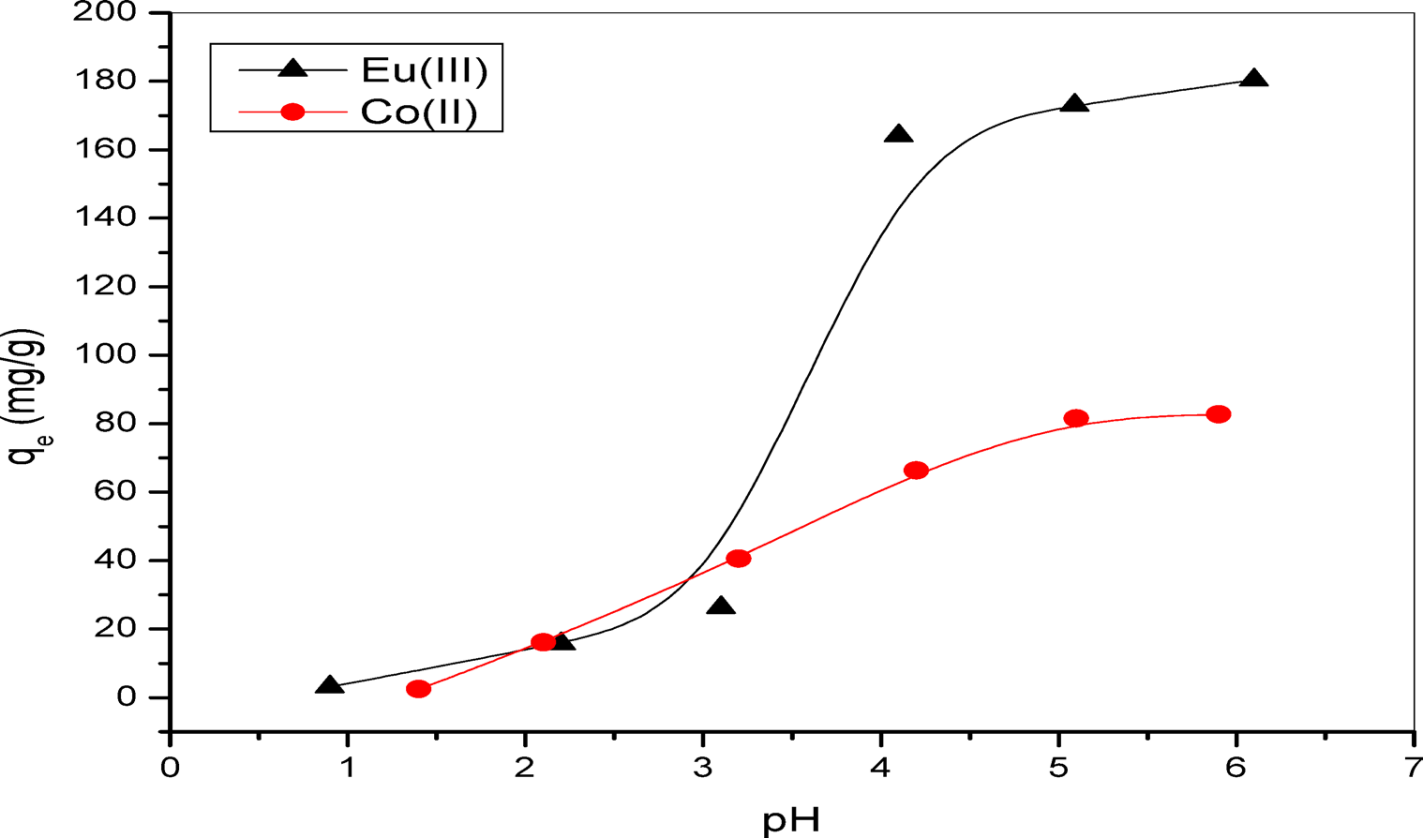
Remediation values of ^152–154^Eu and ^60^Co onto the Alg@N-Fe_3_O_4_@N-Al_2_O_3_@N-Bent nanocomposite at different pH levels.

The initial parameter chosen for this study was the pH effect, aimed at examining the role of hydrogen ions in the remediation process of ^152–154^Eu and ^60^Co radioactive nuclides using the Alg@N-Fe_3_O_4_@N-Al_2_O_3_@N-Bent nanocomposite. This investigation seeks to determine the optimal pH levels for the effective removal of these two radioactive contaminants. In Fig. 5, the adsorption capacities are presented in mg g^−1^ for pH values between 1.0 and 6.0. It is important to note that the removal studies for the radioactive isotopes ^152–154^Eu and ^60^Co at pH values greater than 6.0 were not carried out, owing to the precipitation caused by hydroxide formation^8,34^. The behavior of the remediation process can be categorized into two distinct types. The first type was observed at low pH conditions, where the functional groups present on the surface of the nanocomposite, such as –COOH and –OH, underwent protonation because of the elevated hydrogen ion concentrations. This situation resulted in a competition for the active adsorption sites between H^+^ ions and the radioactive nuclides ^152–154^Eu and ^60^Co^8,34^. Alternatively, by augmenting the pH value, the active sites were rendered accessible for binding with the positively charged radioactive nuclides, ^152–154^Eu and ^60^Co, thus enhancing the efficiency of adsorption^8,34^. The adsorption capacities were found to be at their lowest at pH 1.0, measuring 3.15 mg g^−1^ for ^152–154^Eu and 2.41 mg g^−1^ for ^60^Co radioactive nuclides. Conversely, the most effective removal of these radionuclides was observed at pH 6.0, yielding 180.40 mg g^−1^ for ^152–154^Eu and 82.71 mg g^−1^ for ^60^Co.

**Fig. 6:**
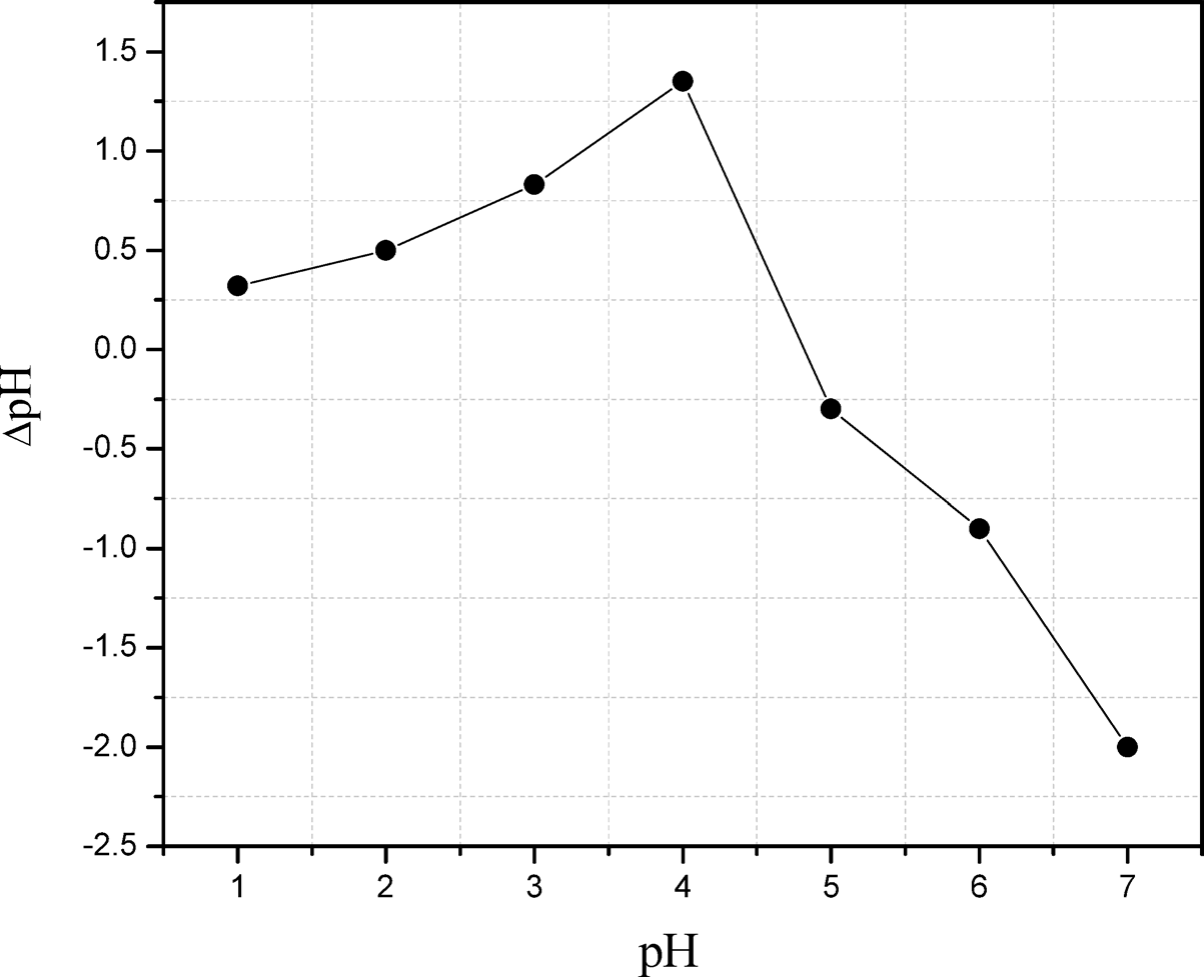
The pH point of zero charge (pH_pzc_) of the Alg@N-Fe_3_O_4_@N-Al_2_O_3_@N-Bent nanocomposite at 25 °C.

These results were validated by determination of the pH point of zero charges (pHpzc) for the synthesized Alg@N-Fe_3_O_4_@N-Al_2_O_3_@N-Bent nanocomposite. As illustrated in Fig. 6, the pH_pzc_ of Alg@N-Fe_3_O_4_@N-Al_2_O_3_@N-Bent was determined to be 4.0. Therefore, at this pH level, the surface of the nanocomposite exhibits neutrality. The finding is presented in Fig. 6 also indicate that Alg@N-Fe_3_O_4_@N-Al_2_O_3_@N-Bent carries a positive charge when the pH is below 4. This clarifies the decline in adsorption capacity under this pH, which could be linked to the strong competition between H^+^ and ^152–154^Eu(III) or ^60^Co(II) ions^16^. When the pH exceeds 4, the active sites on the nanocomposite developed a negative charge as a consequence of the hydrolysis of the composite’s functional groups (–COOH, –OH, etc.). This phenomenon promotes the adsorption of ^152–154^Eu (III) and ^60^Co (II) radionuclide ions, facilitated by electrostatic attraction^17^. Consequently, to prevent the formation of insoluble hydroxides of ^152–154^Eu (III) and ^60^Co (II) at higher pH levels, a pH value of 6 was employed in the subsequent experiments^16^. Additionally, the adsorption capacity is directly related to the ionic potential, which is defined as Z/r of an ion, with Z indicating the charge and r signifying the radius of the ion. This holds particularly true for Eu(III), an f-block element characterized by predominantly electrostatic coordination bonds. Therefore, it is justifiable to assert that ^152–154^Eu(III) possesses a superior adsorption capability in comparison to ^60^Co(II) onto the Alg@N-Fe_3_O_4_@N-Al_2_O_3_@N-Bent nanocomposite^16^. As a result, it can be deduced that a considerable consistency is evident between the outcomes of this test and the studies concerning the effect of initial pH on the removal efficiency ofAlg@N-Fe_3_O_4_@N-Al_2_O_3_@N-Bent.

#### The impact of contact time on the adsorption process

**Fig. 7:**
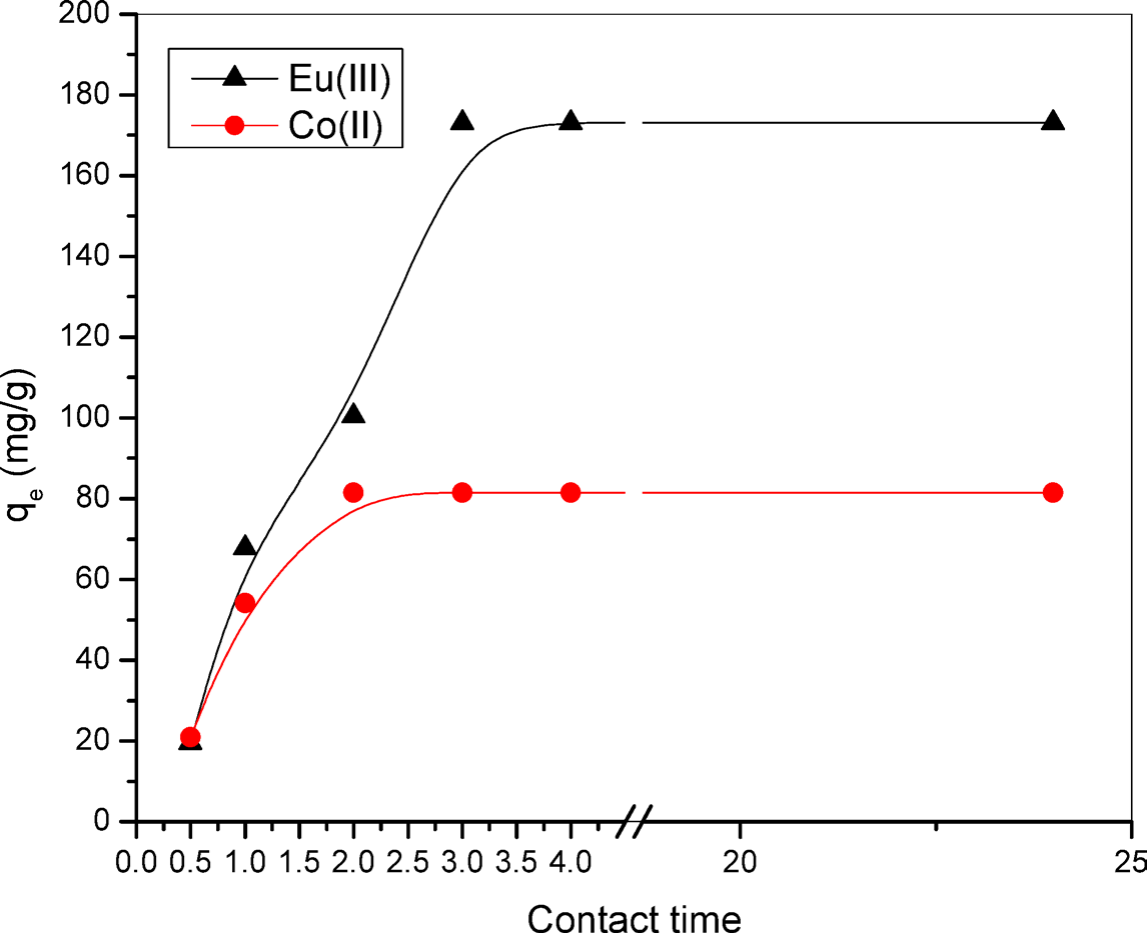
Remediation values of ^152–154^Eu and ^60^Co onto the Alg@N-Fe_3_O_4_@N-Al_2_O_3_@N-Bent nanocomposite at different contact time.

This study investigated the adsorption characteristics of the radioactive nuclides ^152–154^Eu and ^60^Co using the Alg@N-Fe_3_O_4_@N-Al_2_O_3_@N-Bent nanocomposite across various time intervals (0.5, 1.0, 1.5, 2.0, 2.5, 3.0, 3.5, 4.0, and 24.0 min). The optimal pH level for the adsorption of these nuclides was established at 6.0. Figure 7 illustrates the impact of contact time on the adsorption process of ^152–154^Eu and ^60^Co. It was found that the capacity for adsorption rises with extended contact time, ultimately stabilizing as time continues to increase. The adsorption process achieved its equilibrium condition at 3.0 and 2.0 min, with values of 173.12 mg g^−1^ and 81.50 mg g^−1^ for the radioactive nuclides ^152–154^Eu and ^60^Co, respectively. These findings are illustrated in Fig. 7, which presents the relationship between mg g^−1^ and contact time.

The kinetics associated with the adsorption of radioactive nuclides by the Alg@N-Fe_3_O_4_@N-Al_2_O_3_@N-Bent nanocomposite were evaluated through four distinct kinetic models: *pseudo*-first order, Elovich, *pseudo*-second order, and intra-particle diffusion^35,36^. In this context, Eq. (3) illustrates the *pseudo*-first order model, with the slope representing the adsorption rate constant denoting K_1_, obtained from the graph of ln(q_e_ - q_t_) versus time (t)

3 $${\text{ln }}\left( {{{\text{q}}_{\text{e}}} - {{\text{q}}_{\text{t}}}} \right){\text{ }}={\text{ ln}}{{\text{q}}_{\text{e}}} - {\text{ }}{{\text{K}}_{\text{1}}}{\text{t}}~~~$$

Where, q_e_ indicates the amount of radioactive nuclides that have been adsorbed (mg g^−1^) when equilibrium is reached, whereas q_t_ refers to the amount of adsorbed radioactive nuclides (mg g^−1^) at a given time (t) in min. Furthermore, k_1_ is the rate constant associated with the adsorption process, measured in (g/mg min).

The *pseudo*-second order is characterized by Eq. (4), in which the slope, identified as k_2_ (g mg^−1^ min^−1^), functions as the rate constant for the second-order process, as demonstrated in the graph of t/q_t_ against q^2^.

4 $${\text{t}}/{{\text{q}}_{\text{t}}}={\text{ 1}}/{{\text{k}}_{\text{2}}}{{\text{q}}_{\text{e}}}^{{\text{2}}}+{\text{ t/}}{{\text{q}}_{\text{e}}}~$$

According to the intraparticle diffusion model, the adsorption mechanism unfolds in two sequential stages. The first stage encompasses the diffusion of external ^152–154^Eu and ^60^Co radioactive nuclides onto the surface of the Alg@N-Fe_3_O_4_@N-Al_2_O_3_@N-Bent nanocomposite. This is succeeded by a second stage that involves the diffusion of these nuclides into the internal pores of the nanocomposite. The model is represented by the relationship between q_t_ and t_1/2_, where the slope, identified as K_id_ (mg/g min), indicates the rate constant for intra-particle diffusion, as presented in Eq. (4).

5 $${{\text{q}}_{\text{t}}}={\text{ }}{{\text{k}}_{{\text{id}}}}{{\text{t}}^{{\text{1}}/{\text{2}}}}+{\text{ C}}$$

The fourth model, namely Elovich, is expressed by Eq. (6), which posits that the adsorption process is established on a heterogeneous surface, serving as an indication of the chemisorption phenomenon.

6 $${{\text{q}}_{\text{t}}}={\text{ 1}}/\beta {\text{ ln}}\alpha \beta {\text{ }}+{\text{ 1}}/\beta {\text{ lnt}}$$

Where, α represents the initial adsorption rate measured in mg/g min, whereas β denotes the surface coverage of the nanocomposite and is refers to the activation energy associated with chemisorption.

The four models assessed for their ability to characterize the adsorption of ^152–154^Eu and ^60^Co radioactive nuclides onto the Alg@N-Fe_3_O_4_@N-Al_2_O_3_@N-Bent nanocomposite have had their kinetic parameters calculated and presented in Table 3. It was determined that the adsorption of the radioactive nuclide ^152–154^Eu conformed to first-order, second-order, and intra-particle models. In contrast, the adsorption of ^60^Co radioactive nuclides was accurately described by either first-order or second-order models, based on the derived correlation coefficients. According to the findings of the kinetic study, the adsorption of radioactive nuclides was consistent with the *pseudo*-second-order model, with film diffusion being favored as the primary mechanism in comparison to intra-particle diffusion. As a consequence, the surface characteristics of the Alg@N-Fe_3_O_4_@N-Al_2_O_3_@N-Bent nanocomposite may reveal non-homogeneity, as evidenced by the inconsistencies with the Elovich model. This observation suggests a greater likelihood that this reaction proceeds via an ion-pair reaction mechanism, involving the creation of complexes.

**Table 3:** Kinetic parameters for the adsorption of ^152–154^Eu and ^60^Co onto the Alg@N-Fe_3_O_4_@N-Al_2_O_3_@N-Bent nanocomposite.

Kinetic models	Equations	Kinetic parameters	^152–154^Eu	^60^Co
Pseudo-first order	Ln (q_e_−q_t_ ) = Ln (q_e_) −k_1_t	q_e_(mg g^−1^)	401.01	95.87
		K_1_ (min^−1^)	1.233	1.375
		R^2^	0.931	0.972
Pseudo-second order	t/q_t_= 1/k_2_q_e_^2^ + t/q_e_	q_e_(mg g^−1^)	88.33	200
		K_2_ (g mg^−1^min^−1^)	1.15 E10^6^	4.44 E10^6^
		R^2^	0.996	0.974
Intra-particle diffusion	q_t_ = k_id_ t^1/2^ + C	K_id_ (mg g^−1^min^−1/2^)	122.3	44.54
		C	-61.93	1.776
		R^2^	0.957	0.818
Elovich	q_t_ = 1/β Ln(αβ ) + 1/β Ln (t)	α (mg g^−1^min^−1^)	270.68	515.5
		β (mg g^−1^)	0.024	0.070
		R^2^	0.724	0.596

#### The impact of the initial radioactive nuclides concentration on the adsorption process

**Fig. 8:**
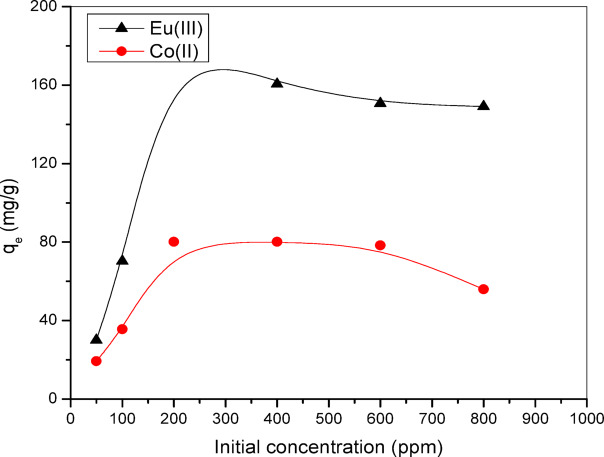
Remediation values of ^152–154^Eu, and ^60^Co onto the Alg@N-Fe_3_O_4_@N-Al_2_O_3_@N-Bent nanocomposite at different initial radioactive nuclides concentration.

The analysis of the initial concentrations of the radioactive nuclides ^152–154^Eu and ^60^Co on the adsorption process, as illustrated in Fig. 8, revealed that adsorption levels rose with higher concentrations of the radioactive nuclides, while a decrease in adsorption was noted at lower concentrations. This behavior was assessed in light of the heightened mass transfer barrier that exists between the adsorbate, which includes the radioactive nuclides ^152–154^Eu or ^60^Co in the solution, and the functional groups present in the Alg@N-Fe_3_O_4_@N-Al_2_O_3_@N-Bent nanosorbent. The minimum adsorption values were recorded at a concentration of 50.0 mg/L, resulting in removal capacities of 19.28 mg g^−1^ for ^60^Co and 30.11 mg g^−1^ for ^152–154^Eu radioactive nuclides. Conversely, the maximum adsorption values were achieved at a concentration of 400.0 mg/L, where the removal capacities for ^60^Co and ^152–154^Eu were mg g^−1^ 80.11 and 160.54 mg g^−1^, respectively.

Four distinct adsorption models were examined to elucidate the various adsorption mechanisms and to evaluate the maximum adsorption capacities of the tested radioactive nuclides. The linear adsorption models, including Langmuir, Freundlich, Temkin, and Dubinin–Radushkevich (D–R), were utilized and examined, allowing for the calculation of the adsorption isotherm parameters^37,38^.

The first model analyzed was Langmuir, where the mechanism of adsorption is characterized as a unimolecular reaction with the potential for reversibility. In this instance, adsorption is facilitated on a monolayer and homogeneous surface of the nanocomposite, as described by the following equation, Eq. (7).

7 $${\text{1}}/{{\text{q}}_{\text{e}}}={\text{ 1}}/{{\text{q}}_{{\text{max}}}}+{\text{1}}/{{\text{q}}_{{\text{max}}}}{{\text{K}}_{\text{L}}}{{\text{C}}_{\text{e}}}$$

The variable q_e_ represents the concentration at equilibrium (mg g^−1^) of the radioactive nuclides ^152–154^Eu and ^60^Co adsorbed onto the Alg@N-Fe_3_O_4_@N-Al_2_O_3_@N-Bent nanocomposite. The term q_max_ denotes the maximum adsorption capacity (mg/g) of these radioactive nuclides on the surface of the nanocomposite. Additionally, C_e_ (mg/L) indicates the concentration at equilibrium for ^152–154^Eu and ^60^Co, while K_L_ (L mg^−1^) refers to the saturation constant.

Freundlich was the second model applied, illustrating that adsorption took place through the interaction between the active binding sites of the adsorbent and the adsorbate on a heterogeneous surface, as represented by either Eq. (8) or Eq. (9).

8 $${{\text{q}}_{\text{e}}}={\text{ }}{{\text{K}}_{\text{F}}}{\text{C}}{{\text{e}}_{\text{}}}/{\text{n}}$$

9 $${\text{ln}}{{\text{q}}_{\text{e}}}={\text{ ln }}{{\text{K}}_{\text{F}}}+{\text{ 1}}/{\text{n ln}}{{\text{C}}_{\text{e}}}$$

Where, q_e_ (mg g^−1^) represents the quantity of adsorbed radioactive nuclides ^152–154^Eu and ^60^Co, while C_e_ denotes the concentration of these nuclides in the liquid phase at equilibrium. Moreover, K_F_ is the Freundlich constant, which reflects the bonding energy associated with the adsorption process.

The third model pertains to Temkin, where the adsorption is influenced by the heat of reaction as outlined in Eq. (10).

10 $${{\text{q}}_{\text{e}}}={\text{ }}\left( {{{\text{R}}_{\text{T}}}/{{\text{b}}_{\text{t}}}} \right){\text{ ln }}{{\text{a}}_{\text{t}}}+{\text{ }}\left( {{{\text{R}}_{\text{T}}}/{{\text{b}}_{\text{t}}}} \right){\text{ln}}{{\text{C}}_{{\text{e}}~~}}$$

Where, the term q_e_ (mg g^−1^) indicates the amount of ^152–154^Eu and ^60^Co radioactive nuclides that have been adsorbed at equilibrium. The parameter b_t_ is identified as the Temkin constant (mg L^−1^), and it represents the equilibrium binding constant (Lg^−1^). Furthermore, (R_T_/b_t_) (J/mol) is defined as the adsorption heat constant.

The final model, referred to as D–R (Dubinin–Radushkevich), involves an adsorption mechanism facilitated by a porous structure, and it evaluates the energy of adsorption based on Eq. (11).

11 $${\text{ln}}{{\text{q}}_{\text{e}}}={\text{ ln}}{{\text{q}}_{\text{s}}} - {\text{ }}\left( {{{\text{K}}_{{\text{ad}}}}{\varepsilon ^{\text{2}}}} \right)~~~$$

The term q_s_ (mg g^−1^) represents the theoretical saturation capacity of the nanosorbent, while K_ad_ (J mol^−1^) denotes the D–R isotherm constant associated with the average free energy per mole, and q_e_ is the measurement of the adsorption capacity of radioactive nuclides ^152–154^Eu and ^60^Co, quantified in milligrams per gram (mg g^−1^). Additionally, Ɛ corresponds to the Polanyi potential in connection with equilibrium, as outlined in Eq. (12).

12 $$\varepsilon {\text{ }}={\text{ RTln }}\left( {{\text{1}}+{\text{ 1}}/{\text{ }}{{\text{C}}_{\text{e}}}} \right)$$

Where, R represents the universal gas constant, with a value of 8.314 J mol^−1^ K^−1^ and T represents the temperature in Kelvin.

The analysis of the correlation coefficients obtained in this study indicates that the adsorption of the radioactive nuclide ^152–154^Eu by the Alg@N-Fe_3_O_4_@N-Al_2_O_3_@N-Bent nanocomposite is comprehensively explained by both the Langmuir and D-R models. On the other hand, the adsorption of the radioactive nuclide ^60^Co is compatible with the Langmuir, Temkin, and D-R models. Finally, Table 4 provides a detailed illustration of the adsorption parameters, highlighting the alignment of radioactive nuclide adsorption with unimolecular chemical adsorption reactions. These reactions take place on the heterogeneous surface of the nanosorbent, characterized by a variety of available sites and distinct adsorption energies, thereby aiding in the removal of radioactive nuclides^37,38^.

**Table 4:** Adsorption ifsotherm parameters for ^152–154^Eu and ^60^Co onto the Alg@N-Fe_3_O_4_@N-Al_2_O_3_@N-Bent nanocomposite.

Adsorption model	Equations	Adsorption parameters	^152–154^Eu	^60^Co
Langmuir	Ce /q_e_=1/q_max_ K_L_+ Ce /q_ma*x*_	q_m_(mg g^−1^)	66.66	0.77
		K_L_ (Lmg^−1)^	9.73 E10^−3^	0.032
		R^2^	0.954	0.904
Freundlich	Ln (qe) = Ln(K_F_) + 1/n Ln(C_e_)	n	0.648	0.648
		K_F_ (Lmg^−1^)	4.66	0.073
		R^2^	0.736	0.785
Temkin	q_e_=(RT/b_T_)Ln(a_T_)+( R_T_/ b_T_)ln (c_e_)	B (j/mol)	10.60	6.132
		a_T_ (Lg^−1^)	0.208	44.18
		R^2^	0.736	0.919
Dubinin–Radushkevich (D–R)	Ln(q_e_) = Ln (q_s_) - (K_ad_ ἑ^2^)	q_s_ (mgg^−1^)	42.01	12.24
		K_ad_ (mol^2^/Kj^2^)	0.001	-9.0 E10^−15^
		R^2^	0.971	0.96

#### The impact of the temperature of reaction on the adsorption process

**Fig. 9:**
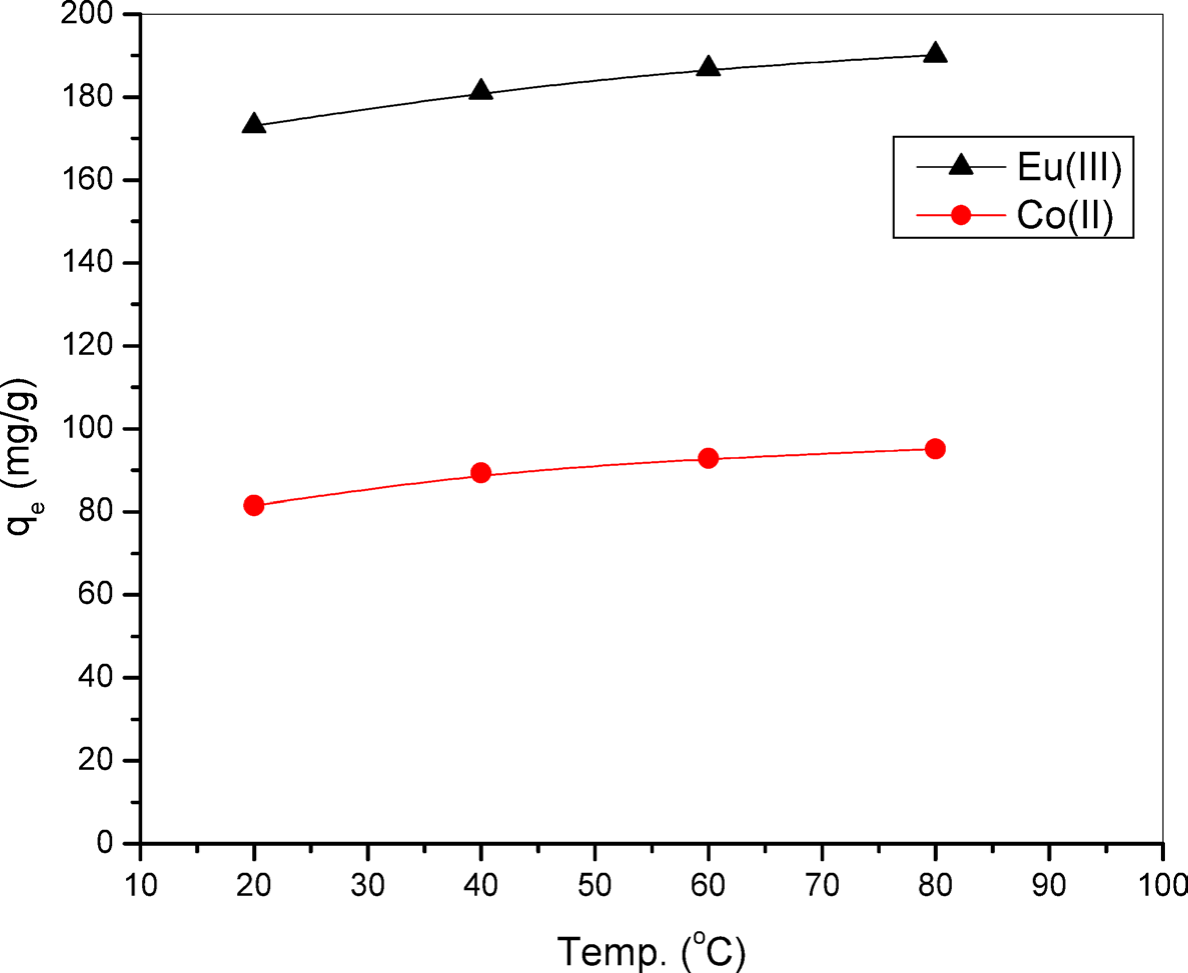
Remediation values of ^152–154^Eu and ^60^Co onto the Alg@N-Fe_3_O_4_@N-Al_2_O_3_@N-Bent nanocomposite at different adsorption temperature.

Figure 9 illustrates the effect of temperature of reaction on the adsorption capacity of the radionuclides ^152–154^Eu(III) and ^60^Co(II) onto the Alg@N-Fe_3_O_4_@N-Al_2_O_3_@N-Bent nanocomposite. It was noted that the values of q_e_ tended to rise with higher adsorption temperatures. This phenomenon is predominantly associated with a reduction in the viscosity of solution mixture, which in turn accelerates the rate of diffusion for radionuclides into the pores of the nanocomposite. Additionally, the breaking of certain internal bonds at the boundaries of the active surface sites of the nanocomposite, along with the greater flexibility of the chains as temperature rises, may contribute to an increase in the number of available active adsorption sites^36^.

## Conclusion

An innovative magnetic nanocomposite was developed through the integration of nanomagnetite, nanoalumina, nanobentonite, and sodium alginate (Alg@N-Fe_3_O_4_@N-Al_2_O_3_@N-Bent). This nanocomposite underwent comprehensive chemical and morphological characterization utilizing various techniques, including FT-IR, TGA, SEM, and XRD. The newly designed Alg@N-Fe_3_O_4_@N-Al_2_O_3_@N-Bent nanocomposite was employed for the adsorptive removal of ^152–154^ Eu and ^60^Co radioactive nuclides. At a pH level of 6.0, the highest adsorption values were achieved, with ^152–154^Eu radioactive nuclides showing an adsorption of 180.40 mg g^−1^, while the ^60^Co radioactive nuclides exhibited an adsorption of 82.71 mg g^−1^. The study revealed that the data of adsorption kinetics were consistent with both first- and second-order models for the radioactive nuclides ^152–154^Eu and ^60^Co, with intra-particle kinetics also being relevant for ^60^Co. The adsorption data were successfully modeled using the Langmuir and Dubinin-Radushkevich (D-R) models for both ^152–154^Eu and ^60^Co, in addition to the Temkin model for ^152–154^Eu. The gathered data affirmed the potential of the Alg@N-Fe_3_O_4_@N-Al_2_O_3_@N-Bent nanocomposite in addressing the remediation of nuclear wastewater contaminated with the radioactive nuclides ^152–154^Eu and ^60^Co, focusing on minimizing their hazards prior to discharge into the environment.

## Data Availability

All data generated or analyzed during this study are included in the submitted files.
